# Click emission in Dall’s porpoise *Phocoenoides dalli*, focusing on physical properties of tissues

**DOI:** 10.1371/journal.pone.0202426

**Published:** 2018-09-14

**Authors:** Mika Kuroda, Motoki Sasaki, Kazutaka Yamada, Nobuhiro Miki, Natsuki Matsui, Takashi Matsuishi

**Affiliations:** 1 Graduate School of Fisheries Sciences, Hokkaido University, Hakodate, Hokkaido, Japan; 2 Department of Veterinary Medicine, Obihiro University of Agriculture and Veterinary Medicine, Obihiro, Hokkaido, Japan; 3 Department of Veterinary Medicine, Azabu University, Sagamihara, Kanagawa, Japan; 4 School of Systems Information Science, Future University Hakodate, Hakodate, Hokkaido, Japan; 5 Global Institution for Collaborative Research and Education, Faculty of Fisheries Sciences, Hokkaido University, Hakodate, Hokkaido, Japan; University of Hong Kong, HONG KONG

## Abstract

Dall’s porpoise (*Phocoenoides dalli*) is one of most common North Pacific porpoise species, for which information on sound-emitting processes is limited. To evaluate the mechanism of click emission in the head of this porpoise, the distribution of acoustic impedance in head tissues was calculated using density and Young’s modulus‘which is a measure of linear resistance to linear compression. Two Dall’s porpoise heads were examined: one for macroscopic dissection, and one for investigating the distribution of acoustic impedance calculated from CT-measured density, and Young’s modulus measured by creep meter. Acoustic impedance increased from the dorsal bursae to the melon’s emitting surface, with impedance matching observed at the boundary between the emitting surface and seawater, and was more similar in distribution to Young’s modulus than it was to density. The distribution of acoustic impedance differed from that of harbor porpoise (*Phocoena phocoena*), despite similarities in the sound-producing organs in the heads of Dall’s and harbor porpoises. A comparison of the physical properties of Dall’s and harbor porpoise head tissues suggests that hypertrophic vestibular sacs and an oval emitting surface are common characteristics in Phocoenidae.

## Introduction

Dall’s porpoise (*Phocoenoides dalli* (True, 1885)) is a small toothed whale found in the North Pacific Ocean and adjacent Bering Sea, Okhotsk Sea and Sea of Japan [1]. Although the diet and distribution of this species are reasonably well known [2–3], there is limited information on sound production in this species. Toothed whales generally produce two types of clicks: narrow band high frequency (NBHF) clicks with a single peak around 130 kHz [4], and wide band (WB) clicks, usually with two moderate peaks around 45 kHz and 110 kHz [5]. Dall’s and the closely related harbor (*Phocoena phocoena*) and finless (*Neophocaena phocaenoides*) porpoises use NBHF clicks [6–8].

Anatomical studies have contributed significantly to our understanding of click-emitting processes. Clicks are produced by trembling of the phonic lips, including two pairs of dorsal bursae [9–10]. Phonic lips and dorsal bursae are located on the rostral and caudal walls of the nasal passage; vibration of the dorsal bursae propagates to the melon, a fatty tissue containing connective tissue, which focuses the “sound beam” with the assistance of air sacs, facial muscles, bone and dense connective tissue (DCT) [9, 11–12]. At the melon’s frontal face the click beam is emitted from a circular aperture, an emitting surface (ES herein, [13]), comprised of DCT [14].

The click-emitting processes of the harbor porpoise and bottlenose dolphin (*Tursiops truncatus*) are well known comparing to Dall’s porpoise. One contributing factor will be that, to the best of our knowledge, Dall’s porpoises have not been maintained continuously in captivity by any institute or aquarium. With the exception of a conventional study [15], wherein differences in sound-producing organs of harbor, Dall’s and finless porpoises were described, anatomical information on the click-emitting processes of Dall’s porpoises is also limited. In addition, more acoustic experiments and anatomical studies have been conducted on harbor porpoise [9, 11, 16] than Dall’s porpoises. Previously we measured acoustic impedance in harbor porpoise head tissues, and compared this to seawater in a study that focused on one characteristic of sound propagation: that when acoustic impedance matched between two objects, sound propagated efficiently [13]. Here, in a similar study, we report the distribution of the acoustic impedance and anatomical structure in the head of Dall’s porpoise.

## Materials and methods

### Specimens

For dissection, computed tomography (CT) imaging, and measurement of physical properties, two adult male Dall’s porpoise carcasses (SNH14026-1,219.5cm and SNH14026-2,207.7cm) were procured. Both animals were retrieved dead, having been accidentally caught in set nets at Rausu, Hokkaido, Japan on 25 June 2014. Heads were severed and stored at −20°C for examination: both specimens were examined by CT; SNH14026-2 was used for density measurement and Young’s modulus scanning, and SNH14026-1 was subsequently fixed in 10% formalin for macroscopic dissection.

### Measurement of physical properties

For estimating Young’s modulus and tissue density, CT images were obtained at 1 mm intervals using a multi-slice CT scanner (Asteion TM Super 4 Edition; Toshiba, Tokyo, Japan); for SNH14026-1 and SNH14026-2, helical pitches were 0.5mm and 1mm, and the number of CT images were 709 and 321, respectively. Tube voltage was 120 kV, and tube current 150mA. After scanning, CT values [17] were obtained from CT data. The standard format of CT data was DICOM (Digital Imaging and Communications in Medicine) and a specialized workstation (ZioTerm™ 2009; Ziosoft, Tokyo, Japan) were used for viewing DICOM files. A CT value was determined for each measured point on Young’s modulus that matched the CT image and sliced head photographs. According to conventional study [18], approximation of density (*ρ*) from CT value (*U*) (Eq 1) was proposed and inspected as below.

ρ=U×1000+1 (1)

Young’s modulus, also called as longitudinal elastic modulus is a measure of linear resistance to linear compression and defined as the slope of the linear region of the stress-strain diagram. measurement was performed following our previous study [13]. Young’s modulus and density were measured from 15 head slices of average thickness 10.97 mm (±0.86 mm SD). A creep meter (YAMADEN, RE2-33005C, RE-3305) with column-shaped plunger (φ 5mm), an equipment mainly used to measure the texture of food such as hardness and viscosity, was used to measure Young’s modulus. Immediately prior to measurement, sliced frozen samples were heated at 25°C on a thermostatic stage.

Acoustic impedance (*Z*) was calculated from Young’s modulus (*E*) and tissue density using (*ρ*) using:

$$Z=\sqrt{\rho E}$$ (2)

Physical properties of Young’s modulus were plotted at measured points on a three-dimensional reconstructed CT image. Physical properties of blubber could not be measured in all slices as the area occupied by it was sometimes too small.

Macroscopic dissection for visually confirming the structure of the head structure was conducted by using fixed head of No. SNH14026-1 with 10% formalin solution and unfixed sliced heads of No. SNH14026-2 used for Young’s modulus measurement. Sizing of ES and VS was conducted on Ziocube, a work station for CT images. ES was defined as a region with a CT value of 0 or less in front of the head.

## Results

### Head structure

**Fig 1:**
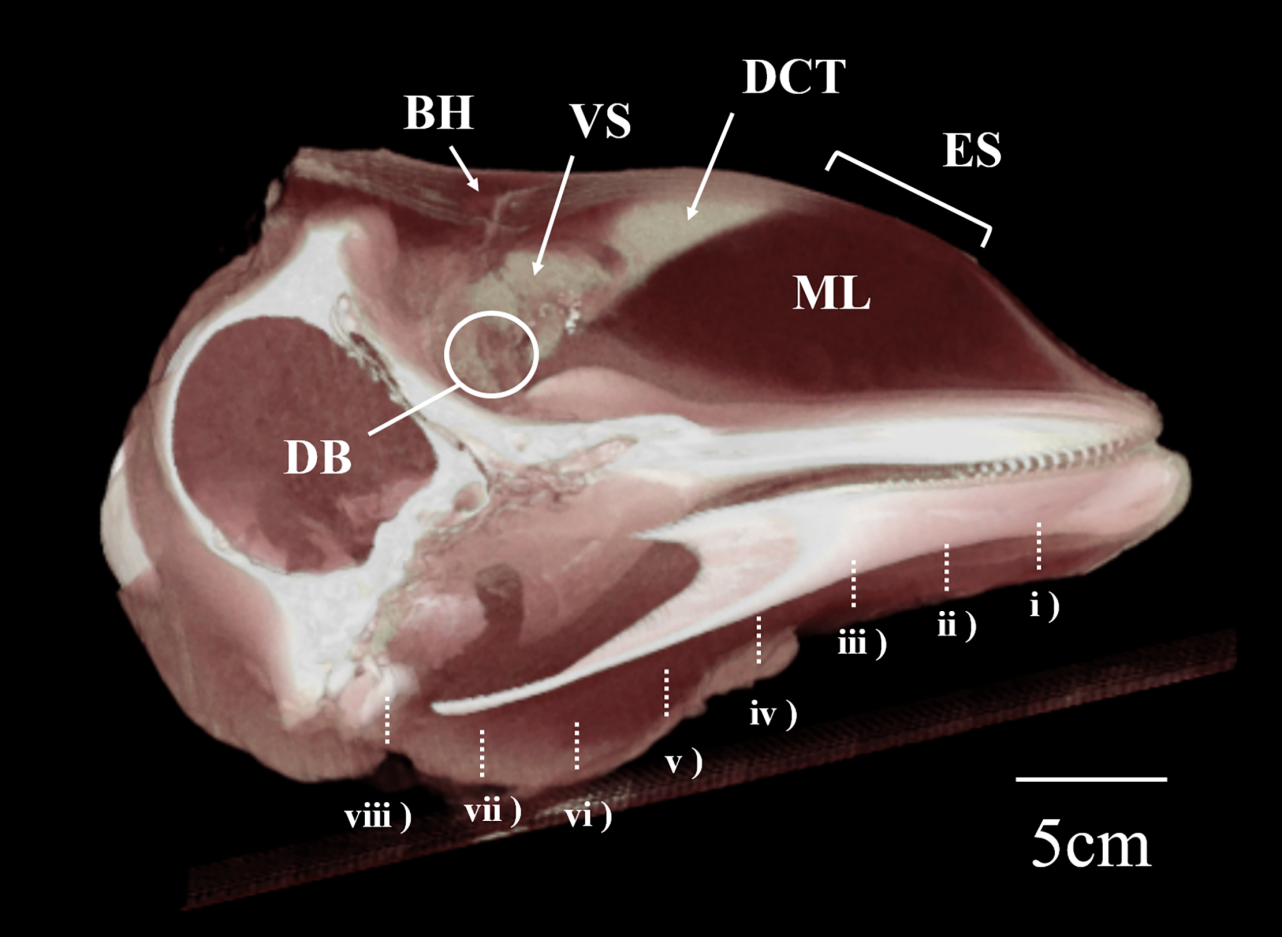
Three-dimensional CT image of longitudinal-sectioned Dall’s porpoise head, SNH14026-1. BH (blowhole), DB (dorsal bursae), VS (vestibular sacs), DCT (dense connective tissue), ML (melon), ES (emitting surface); numbers i–viii correspond to Fig 2 and indicate positions of transverse slices.

**Fig 2:**
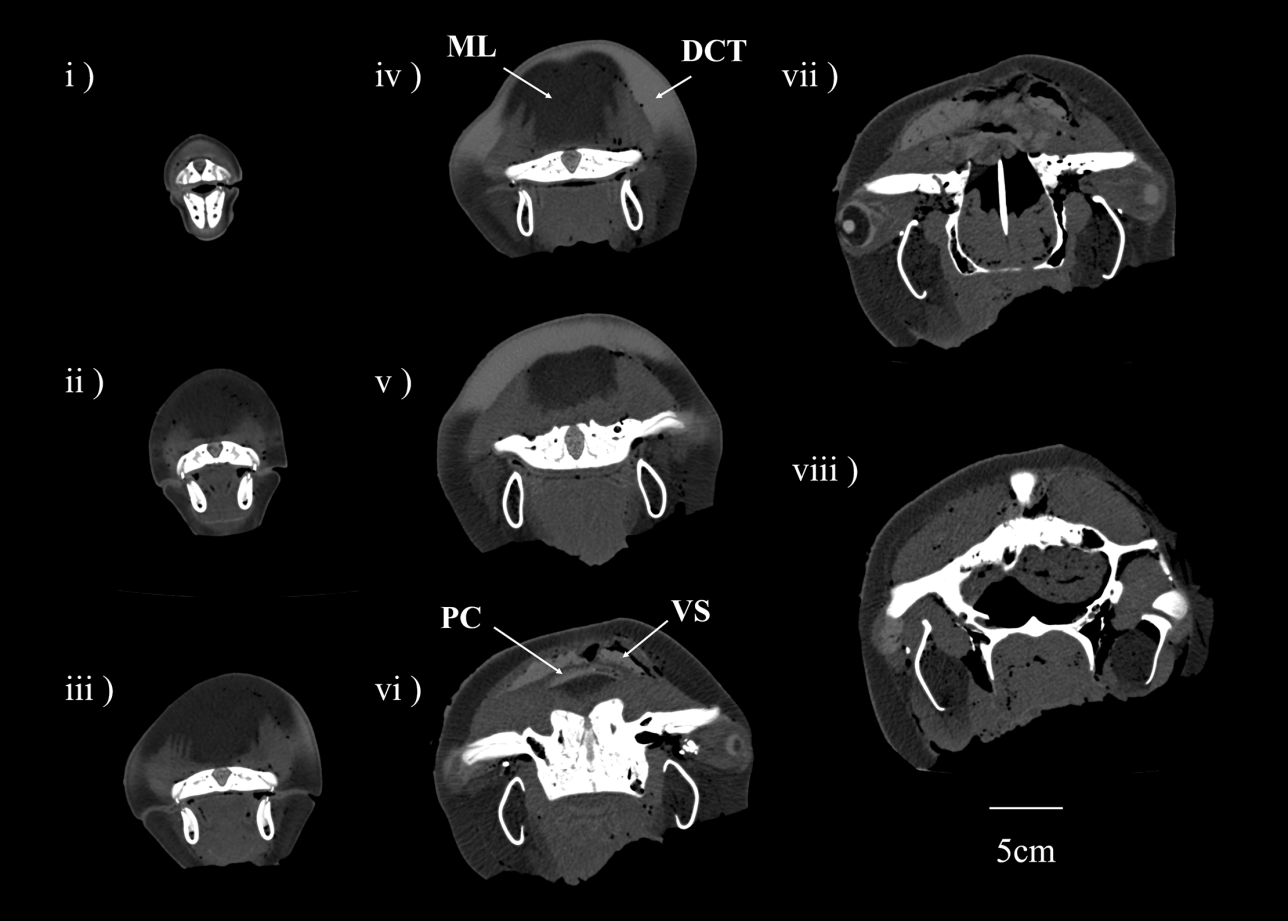
Cross-sectional images of Dall’s porpoise head, SNH-14026-1. ML (melon), DCT (dense connective tissue), VS (vestibular sacs), PC (porpoise capsule); numbers i–viii correspond to numbers in Fig 1.

A reconstructed three-dimensional CT image of a longitudinal-sectioned head, and series of two-dimensional CT images of SNH14026-1 are presented in Figs 1 and 2, respectively. Both figures are based on DICOM files submitted as S1 File and S2 File (see online Supporting Information files). The ES, formed by the DCT and melon, was indicated oval shape and located directly under the skin. Vestibular sacs (VS) indicated in Figs 1 and 2 were hypertrophied, with several thick folded structures. The maximum rostral-caudal length, dorsal-ventral thickness and lateral width of both VS (mm) were 37.3, 17.1 and 46.0 in left VS and 62.5, 17.6 and 54.9 in the right VS.

### Tissue physical properties

**Fig 3:**
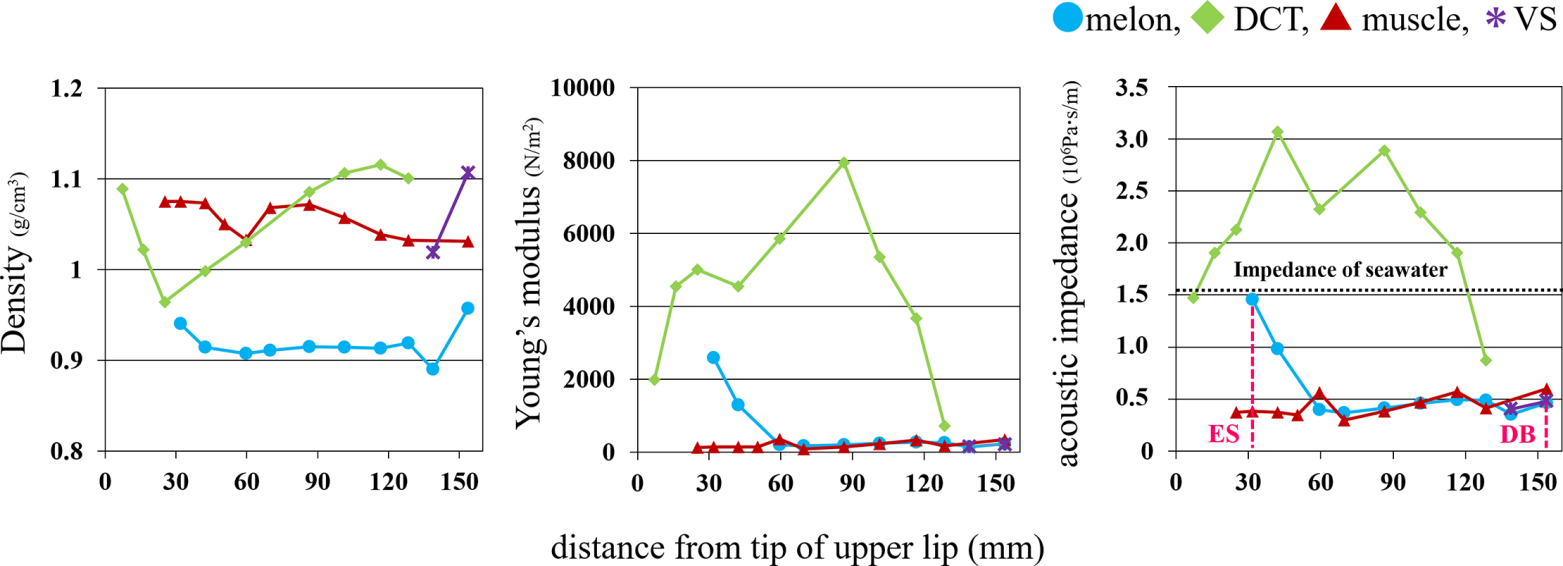
Distribution of average density, Young’s modulus, and acoustic impedance in Dall’s porpoise. Horizontal dotted line (acoustic impedance) indicates seawater impedance, and the vertical broken line the frontal tip of the ES (emitting surface) and DB (dorsal bursae).

Average density, Young’s modulus and acoustic impedance relative to distance from the rostrum for melon, dense connective tissue, blubber and air sacs, and distance from the tip of the upper lip to ES and dorsal bursae (DB, *x* = mm) are depicted in Fig 3. Data source of Fig 3 was submitted as S3 File (see online Supporting Information files). Melon impedance was relatively stable at 5.0×10^6^ Pa s/m from *x* = 59.47mm to 153.67mm, and increased to 1.454×10^6^ Pa s/m at *x* = 31.79mm.

## Discussion

At the ES frontal tip (*x* = 31.79mm), the melon acoustic impedance was 1.454×10^6^ Pa s/m (Fig 3), closely matching that of seawater, 1.54×10^6^ Pa s/m [19]). This result supports the theory that the melon functions to match the acoustic impedance of seawater for the effective emission of clicks [13–14, 20]. The distribution of acoustic impedance was more similar to Young’s modulus than it was to density. It should be caused by the extremely large difference between the density and Young’s modulus (for example, in DCT, the rate of change of Young’s modulus fluctuated from 400–8000N/m^2^, whereas that of density ranged 0.8–1.2 g/cm^3^). Conventional study [13] suggested that focusing distribution of Young's modulus is more important than that of density because when the acoustic impedance changes, the change rate of Young’s modulus would occupy a larger percentage than that of the density. In this study, we supported this idea.

There was little difference in impedance in Dall’s porpoise melon tissues between 60mm and 120mm from the upper lip tip. This is a personal opinion based on the macroscopic observation of the tissue, but the muscles in the lower part of the melon of Dall’s porpoise was assumed to be richer in lipid than that of harbor porpoise [13]. Dall’s porpoise nasal plug muscle tissue appeared to contain more lipid than that of the harbor porpoise, so might have a lower Young’s modulus than other muscle, decreasing the Young’s modulus of melon tissue in contact with this muscle. This anatomical feature was not observed in harbor porpoise [13]. This opinion is not based on quantitative measurement, but since the difference of lipid content can influence the difference in Young’s modulus, it may be the cause of the low Young's modulus indicated by the muscle of dolphin.

In Dall's porpoise and harbor porpoise, the numerical values of Young’s modulus were particularly different in DCT. However, in this study, it was not possible to clarify why this numerical difference occurred and why such a difference was seen in DCT.

It is difficult to relate these differences in acoustic impedance to clicks properties such as source pressure level and / or frequency characteristics. Dall’s and Harbor Porpoise use narrowband and high frequency (NBHF) clicks in common and have similar click spectrograms [6]. However, it is difficult to discuss about similarities and differences in acoustic properties of NBHF clicks in this study because both Dall’s and harbor porpoise are Phocoenidae, while NBHF clicks are reported in various taxa [21].

The anatomy of the Dall’s porpoise (Figs 1 and 2) head and vestibular sacs (VS, Fig 1) were similar to those described by conventional study [15] for harbor porpoise. Despite similarities in the head sizes of these two porpoises, considerable differences in their ES area (59.1cm^2^ and 35.5cm^2^, respectively) exist. Ours is, as far as we are aware, the first account of such differences in ES size for these species. Similarities in VS structure and differences in ES size might be related to characteristic properties of acoustic clicks [15, 22]. In the posterior region of the melon below the VS, some DCT covered dorsal melon surfaces (Figs 1 and 2). We refer to this structure as a “porpoise capsule,” previously described in harbor porpoise [11].

Dall’s porpoise is a fast-swimming coastal-to-offshore species that forms groups of 10 individuals or less, whereas the harbor porpoise lives in coastal areas, and is solitary or typically forms groups of several individuals only. Despite these differences in ecological and social habits, their sound-producing organs are similar in having hypertrophic VS and an oval ES. As a conventional study [15] also reported head structures of Dall’s and harbor porpoises to be similar, hypertrophic VS and an oval ES might be common characteristics in the Phocoenidae.

Our method and results compared the sound-emitting process of Dall’s with harbor porpoises, and considerably improved our understanding of the physical properties of Dall’s porpoise head tissues. We consider the structure of sound propagating organs, and the click-emitting process of Dall’s porpoise to be similar to those of the harbor porpoise. This information improves our understanding of relationships between sound-propagating organs and the acoustic properties of clicks in these small-bodied toothed whales, and perhaps, as a generalization, throughout the family Phocoenidae.

## Supporting information

S1 File Three-dimentional CT data of the head of Dall’s porpoise. CT data of SNH14026-1. File format is DICOM (.dcm).
(DCM)

S2 File Eight CT image data of Dall’s porpoise SNH14026-1. Each CT image data i)—viii) indicates the surface of slice used for Young’s modulus measurement. Additional information for setting of CT scanner was added in the text file, named “S2 Supporting information.txt”.
(ZIP)

S3 File Data sheet for calculating the acoustic impedance. All calculations and graphs were shown in sheet 1.
(XLSX)

## References

[1] Jefferson TA. Dall’s Porpoise *Phocoenoides dalli* In: Perrin WF, Würsig B and Thewissen JGM, editors. Encyclopedia of Marine Mammals second edition. San Diego: Academic press; 2009 p. 296.

[2] Miyashita T, Kasuya T. Distribution and abundance of Dall’s porpoises off Japan. Sci. Rep. Whales Res. Inst., 1988; 39: 121–150.

[3] Ohizumi H, Kuramochi T, Kubodera T, Yoshioka M, Miyazaki N. Feeding habits of Dall’s porpoises (*Phocoenoides dalli*) in the subarctic North Pacific and the Bering Sea basin and the impact of predation on mesopelagic micronekton. Deep-Sea Research I, 2003; 50: 593–610.

[4] Au WWL. Sonar of Dolphins New York: Springer; 1993 p. 104.

[5] Au WWL, Herzing DL. Echolocation signals of wild Atlantic spotted dolphin (*Stenella frontalis*). J. Acoust. Soc. Am. 2003; 113: 598 12558295 10.1121/1.1518980

[6] Kyhn LA, Tougaard J, Beedholm K, Jensen FH, Ashe E, Williams R et al Clicking in a killer whale habitat: Narrow-band, high-frequency biosonar clicks of harbour porpoise (*Phocoena phocoena*) and Dall’s porpoise (*Phocoenoides dalli*). PLOS ONE 8(5): e63763 10.1371/journal.pone.0063763 23723996 PMC3665716

[7] Schevill WE, Watkins WA, Ray C. Click structure in the porpoise, *Phocoena phocoena*. Journal of Mammalogy. 1969; 50(4): 721–728,

[8] Kamminga C, Cohen S, Silber GK. Investigations on cetacean sonar XI: Intrinsic comparison of the wave shapes of some members of the Phocoenidae family. Aquat. Mamm. 1996; 22: 45–55.

[9] Cranford TW, Amundin M, Norris KS. Functional morphology and homology in the odontocete nasal complex: implications for sound generation. J. Morp. 1996; 228: 223–285. 10.1002/(SICI)1097-4687(199606)228:3<223::AID-JMOR1>3.0.CO;2-3 8622183

[10] Aroyan JL, Cranford TW, Kent J, Norris KS. Computer modeling of acoustic beam formation in Delphinus delphis. J. Acoust. Soc. Am. 1992; 92: 2539–2545.

[11] Huggenberger S, Rauschmann MA, Vogl TJ, Oelschläger HH. Functional morphology of the nasal complex in the harbor porpoise (*Phocoena phocoena* L.). Anat. Rec. 2009; 292: 902–920. 10.1002/ar.20854 19306438

[12] McKenna MF, Cranford TW, Berta A, Pyenson ND. Morphology of the odontocete melon and its implications for acoustic function. Mar. Mamm. Sci. 2012; 28: 690–713.

[13] Kuroda M, Sasaki M, Yamada K, Miki N, Matsuishi T. Tissue physical property of the harbor porpoise *Phocoena phocoena* for investigation of the sound emission process. J. Acoust. Soc. Am. 2016; 138: 1451–1456. 10.1121/1.4928608 26428782

[14] Norris KS, Harvey GW. Sound transmission in the porpoise head. J. Acoust. Soc. Am. 1974; 56: 659–664. 4415842 10.1121/1.1903305

[15] Curry BE. Facial anatomy and potential function of facial structures for sound production in the harbor porpoise (*Phocoena phocoena*) and Dall’s porpoise (*Phocoenoides dalli*). Canadian Journal of Zoology. 1992; 70(11): 2103–2114.

[16] Au WWL, Kastelein RA, Benoit–Bird KJ, Cranford TW, McKenna MF. Acoustic radiation from the head of echolocating harbor porpoises (*Phocoena phocoena*). J. Exp. Biol. 2006; 209: 2726–2733. 10.1242/jeb.02306 16809463

[17] Robb RA. Biomedical imaging, visualization, and analysis New York: John Wiley & Sons, Inc.; 1999 p. 33.

[18] Hubbell JH and Seltzer MS. Tables of x-ray mass attenuation coefficients and mass energy-absorption coefficients 1 keV to 20 meV for elements z = 1 to 92 and 48 additional substances of dosimetric interest. National Institute of Standards and Technology. 1995; NISTIR-5632.

[19] National Astronomical Observatory of Japan. Chronological Scientific Tables 2013 Tokyo: Maruzen publishing Co. Ltd.; 2012 p. 430.

[20] Cranford TW, Amundin M. Biosonar pulse production in odontocetes: the state of our knowledge In: Thomas JA, Moss CF, Vater M, editors. Echolocation in bats and dolphins. Chicago: The University of Chicago Press; 2003 pp. 27–35.

[21] Morisaka T, Connor RC. Predation by killer whales (*Orcinus orca*) and the evolution of whistle loss and narrow-band high frequency clicks in odontocetes. J. Evol. Biol. 2007; 20:1439–1458. 10.1111/j.1420-9101.2007.01336.x 17584238

[22] Nakamura K, Tadasu KY, Shimazaki K. Measurements of the nasal sacs of individual common dolphin, *Delphinus delphis*, and Dall’s porpoise, *Phocoenoides dalli*, by means of silicon reconstruction. Mammal Study. 1998; 23: 119–122.

